# A narrative review of PCSK9 inhibitors: from evolocumab to novel discoveries

**DOI:** 10.3389/fphar.2026.1800502

**Published:** 2026-05-21

**Authors:** Hui-Hui Liu, Sha Li, Jian-Jun Li

**Affiliations:** 1 State Key Laboratory of Cardiovascular Disease, Fuwai Hospital, Heart Failure Center, National Center for Cardiovascular Diseases, Chinese Academy of Medical Sciences and Peking Union Medical College, Beijing, China; 2 State Key Laboratory of Cardiovascular Disease, Fuwai Hospital, Cardiometabolic Center, National Center for Cardiovascular Diseases, Chinese Academy of Medical Sciences and Peking Union Medical College, Beijing, China

**Keywords:** evolocumab, inhibitor, mechanism, pleiotropic effect, proprotein convertase subtilisin/kexin type 9

## Abstract

The application of proprotein convertase subtilisin/kexin type 9 (PCSK9) inhibitors represents a crucial milestone in the field of lipid-lowering therapy. As the first PCSK9 inhibitor to be approved globally, evolocumab has not only offered an entirely novel therapeutic option for patients with familial hypercholesterolemia but also established a paradigm for the development of subsequent anti-PCSK9 monoclonal antibodies. This narrative review provides an overview of the discovery of PCSK9 and its role in low-density lipoprotein cholesterol metabolism, detailing the developmental trajectory and key clinical trials of evolocumab, and underscoring its central importance and transformative impact on lipid-lowering therapeutics. On this basis, we conduct a comparative analysis of the differences in mechanism of action, clinical efficacy and safety among the marketed PCSK9 inhibitors, and dissect the limitations and unmet needs in current clinical practice. Furthermore, this review explores the pleiotropic effects of PCSK9 beyond lipid metabolism, and comments on its potential application value and latest research advances in diseases such as infection, liver disease, and malignancy. Finally, we prospect the future development directions of PCSK9-targeted therapy, aiming to provide an integrated reference perspective for basic research and clinical practice in this field.

## 1 Introduction

Cardiovascular disease (CVD) remains the leading cause of death worldwide, resulting in approximately 19.41 million deaths in 2021 (Martin et al., 2025). Dyslipidemia, particularly elevated low-density lipoprotein cholesterol (LDL-C), is a key driver of atherosclerotic CVD (ASCVD) and plays a central role in the initiation and progression of atherosclerosis (Baigent et al., 2010). Substantial evidence indicates that each 1.0 mmol/L reduction in LDL-C is associated with a 20%–25% decrease in cardiovascular event risk (Baigent et al., 2010), underscoring the importance of effective LDL-C lowering in the primary and secondary prevention of ASCVD (Li et al., 2023).

Statins have long been the cornerstone of LDL-C-lowering therapy, effectively reducing circulating LDL-C levels (Li et al., 2023). Beyond their lipid-lowering action, statins exert multiple pleiotropic benefits, including improved endothelial function and vascular tone, anti-inflammatory and antioxidative effects, reduced thrombogenicity, and stabilization of atherosclerotic plaques (German and Liao, 2023). However, statin therapy is also associated with adverse effects such as hepatotoxicity, statin-associated muscle symptom, and an elevated risk of new-onset diabetes (Li et al., 2023). Clinically, muscle-related symptoms affect approximately 10%–30% of patients on statin treatment (Urina-Jassir et al., 2021). Furthermore, even among patients who tolerate statins well, a considerable proportion fail to attain LDL-C targets, leading to persistent residual cardiovascular risk (Mach et al., 2020). Notably, in very-high-risk ASCVD populations, only about 20% achieve the guideline-recommended LDL-C goal of <55 mg/dL (Mach et al., 2020). For patients who are statin-intolerant or unable to reach LDL-C goals with statins alone, additional lipid-lowering strategies are needed—underscoring the demand for novel therapeutic approaches (Lin et al., 2018).

Recent advances in our understanding of atherosclerotic pathogenesis have facilitated the identification of novel therapeutic targets for cholesterol management (Yaghoubi and Sallam, 2025). Among these, proprotein convertase subtilisin/kexin type 9 (PCSK9) plays a crucial regulatory role in cholesterol metabolism and has emerged as a significant target for lipid-lowering interventions (Lin et al., 2018).

Based on a selective literature search of PubMed and Web of Science databases for articles published up to March 2026, using keywords including “PCSK9”, “PCSK9 inhibitor”, “evolocumab”, “alirocumab”, “cardiovascular disease”, “atherosclerosis”, and “clinical trial”, and including peer-reviewed original articles, systematic reviews, meta-analyses, clinical guidelines, and other relevant literature, this narrative review focuses on advances in PCSK9 inhibitors from evolocumab to novel discoveries and discusses their pleiotropic effects and future clinical directions.

## 2 Landscape of PCSK9

### 2.1 Discovery of PCSK9

**FIGURE 1:**
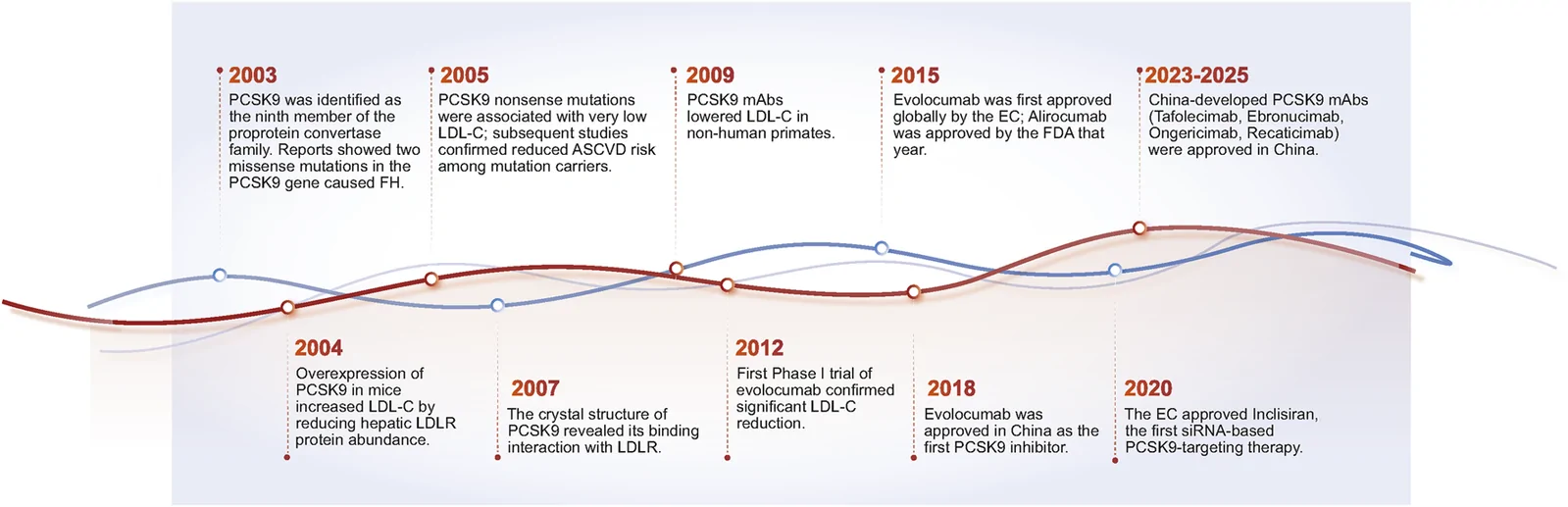
PCSK9 discovery and time point in its pharmaceutical development process PCSK9, proprotein convertase subtilisin/kexin type 9; FH, familial hypercholesterolemia; LDL-C, low-density lipoprotein cholesterol; LDLR, low-density lipoprotein receptor; ASCVD, atherosclerotic cardiovascular disease; mAbs, monoclonal antibodies; EC, European Commission; FDA, U.S. Food and Drug Administration.

PCSK9 was identified approximately 2 decades ago (Barale et al., 2021). In 2003, Abifadel et al. studied a French cohort of families with autosomal dominant familial hypercholesterolemia (FH), characterized by markedly elevated LDL-C levels. Although two major FH-associated genes were known at the time, no pathogenic variants were detected in these families, prompting the search for an additional causative gene. Genetic analysis later revealed that gain-of-function (GOF) mutations in PCSK9 explained the severe hypercholesterolemia in these patients, establishing PCSK9 as the third gene linked to FH (Abifadel et al., 2003). The following years further elucidated the role of PCSK9 in lipid metabolism. In 2004, mouse studies showed that hepatic overexpression of PCSK9 increased circulating LDL-C and reduced hepatic LDL receptor (LDLR) expression (Maxwell and Breslow, 2004). In 2005, data from the Dallas Heart Study demonstrated that loss-of-function (LOF) mutations in PCSK9 were associated with significantly lower LDL-C levels and a markedly reduced risk of ASCVD. Remarkably, individuals with PCSK9 deficiency exhibited serum LDL-C concentrations as low as 14 mg/dL (Cohen et al., 2006). Together, these findings positioned PCSK9 as a promising therapeutic target for lipid-lowering interventions. The determination of the PCSK9 crystal structure in 2007 provided a structural basis for rational drug design (Piper et al., 2007). Building on these foundational discoveries, the development of PCSK9-targeted therapies has advanced through several key milestones, summarized in Figure 1. This timeline outlines pivotal advances in PCSK9 research and clinical translation from 2003 to 2025.

### 2.2 Role of PCSK9 on LDL-C metabolism

**FIGURE 2:**
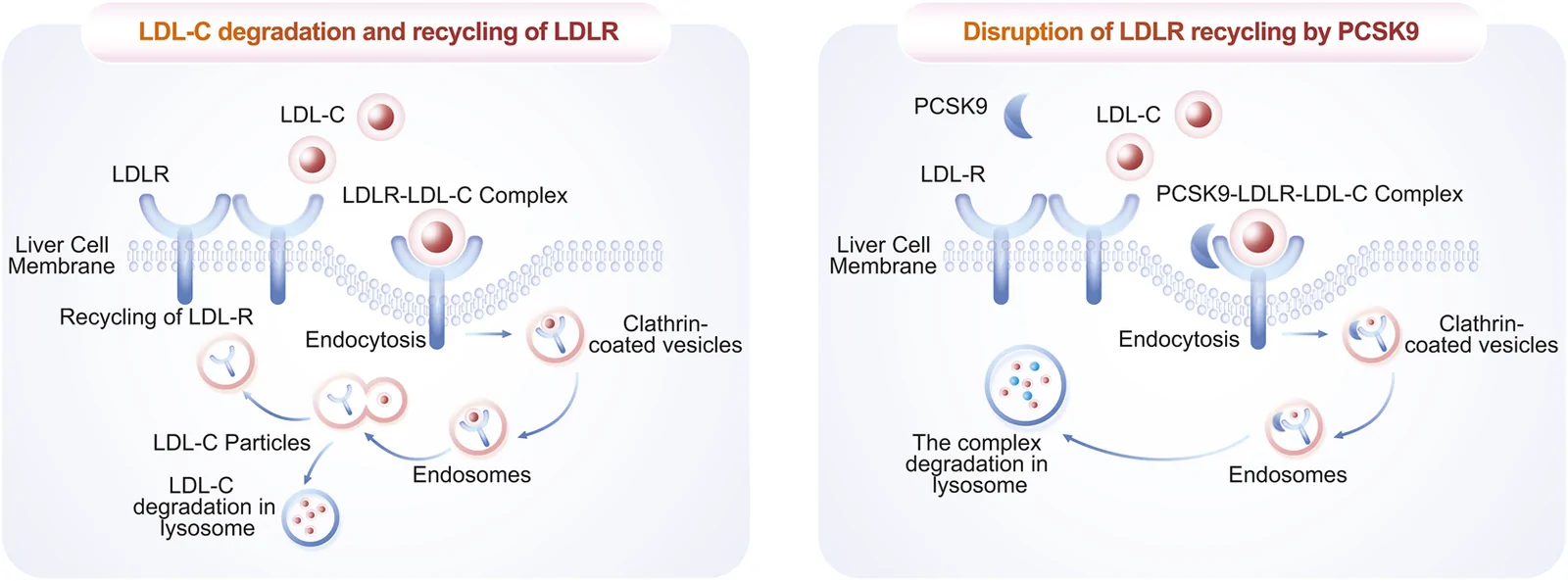
Mechanism of PCSK9 in regulating of LDL-C metabolism. The physiological degradation of LDL-C and recycling of LDLR (left panel), and the disruption of this recycling by PCSK9 (right panel). LDL-C, Low-density lipoprotein cholesterol; LDLR, LDL receptors; PCSK9, Proprotein convertase subtilisin/kexin type 9.

PCSK9 is primarily secreted by hepatocytes and modulates circulating LDL-C levels by binding to the LDLR. Expressed on the hepatocyte surface, LDLR captures circulating LDL-C particles to form an LDLR–LDL-C complex. This complex undergoes endocytosis, and the resulting vesicles fuse with endosomes. Within the acidic endosomal environment, LDL-C dissociates from LDLR and is subsequently transported to lysosomes for degradation into free cholesterol, fatty acids, and amino acids. Under normal conditions, LDLR is recycled back to the hepatocyte membrane to facilitate further LDL-C clearance. However, when PCSK9 binds to LDLR, it induces conformational changes that prevent receptor recycling. Instead, the PCSK9–LDLR–LDL-C complex is diverted to lysosomes for degradation. This process reduces the number of LDLRs available on the hepatocyte surface, thereby impairing LDL-C clearance and elevating circulating LDL-C levels. Consequently, pharmacological inhibition of PCSK9 increases surface LDLR expression, enhances hepatic uptake of LDL-C, and effectively lowers plasma LDL-C concentrations (Lagace et al., 2006) (Figure 2).

## 3 Evolocumab: the first globally approved PCSK9 inhibitor

### 3.1 The development of evolocumab

Following elucidation of the structure and biological function of PCSK9, its inhibition was recognized as a promising strategy for lowering LDL-C levels. Due to the considerable challenge of designing small molecules or peptide mimetics that effectively block the PCSK9–LDLR interaction, monoclonal antibodies targeting circulating PCSK9 emerged as the most clinically feasible approach.

Chan et al. were the first to successfully develop a fully human monoclonal antibody targeting PCSK9 (Chan et al., 2009). This antibody exhibited high affinity for a specific PCSK9 epitope, effectively blocking its interaction with the LDLR and thereby inhibiting PCSK9-mediated lysosomal degradation of LDLR. Preclinical studies demonstrated that the antibody significantly lowered cholesterol levels in both murine and non-human primate models, while showing no lipid-lowering effect in LDLR-knockout mice—confirming that its efficacy depends entirely on the LDLR pathway (Chan et al., 2009). In cynomolgus monkeys, a single administration reduced circulating LDL-C levels by approximately 80% from baseline.

Evolocumab was the first PCSK9 inhibitor to receive regulatory approval for clinical use worldwide (approved by the EU in 17 July 2015). It was generated using the Amgen XenoMouse® platform, which introduces human immunoglobulin genes into mice, thereby enabling the production of fully human, mature monoclonal antibodies. This technology accelerated antibody development, reduced immunogenicity, and enhanced both safety and therapeutic efficacy (Mendez et al., 1997; Yang et al., 2001). However, related research, development, and production processes require substantial financial investment and advanced technical support.

Moreover, evolocumab’s rational molecular design was critical: it employs an IgG2 isotype whose Fc region has low affinity for Fcγ receptors, thereby minimizing unwanted effector functions such as antibody-dependent cellular cytotoxicity (ADCC) and complement-dependent cytotoxicity (CDC). This supports IgG2 as the preferred isotype for PCSK9-targeted antibody design. Furthermore, its fully human nature contributed to exceptionally low immunogenicity, with only 0.3% of patients (48 of 17,992) developing anti-drug antibodies in large clinical trials, and no neutralizing antibodies detected (European Medicines Agency, 2015). Collectively, the success of evolocumab thus established a practical and transferable model that has informed the design and optimization of subsequent PCSK9 monoclonal antibodies.

### 3.2 Key clinical trials of evolocumab

#### 3.2.1 The pioneering first-in-human study of PCSK9 inhibitor

In 2010, evolocumab entered its first phase 1 clinical trial (NCT01133522) to evaluate the pharmacokinetics, efficacy, and safety of this PCSK9 inhibitor in humans. The results were published in 2012 and confirmed that evolocumab significantly reduced circulating free PCSK9 and lowered LDL-C in a dose-dependent manner. A single subcutaneous dose of 420 mg lowered LDL-C by up to 67% from baseline, while repeated weekly administration achieved reductions of up to 81%. The reduction in LDL-C was directly correlated with a dose-dependent decrease in free PCSK9 levels and a corresponding increase in hepatic LDL receptor levels. This precisely confirmed the intended mechanism of action in humans: inhibiting PCSK9 prevents LDL receptor degradation, leading to enhanced clearance of LDL-C from the blood. The incidence of adverse events was comparable to placebo, and no serious safety concerns emerged (Dias et al., 2012). This trial marked the successful translation of PCSK9 biology into human therapeutics, validating PCSK9 inhibition as a clinically viable strategy and providing the foundational rationale for subsequent PCSK9 inhibitor development.

#### 3.2.2 GLAGOV: the first to confirm coronary plaque regression by PCSK9 inhibitor

The GLAGOV trial (Nicholls et al., 2016) was the first imaging-based clinical trial to assess the effects of a PCSK9 inhibitor on coronary atherosclerotic plaque burden. In this multicenter, double-blind, placebo-controlled study, 968 patients with prior coronary angiography were randomized (1:1) to receive subcutaneous evolocumab 420 mg monthly or placebo for 76 weeks. The primary endpoint was the change in percent atheroma volume (PAV), measured by serial intravascular ultrasound (IVUS), from baseline to week 78.

The results demonstrated that adding evolocumab to intensive statin therapy significantly promoted plaque regression (Nicholls et al., 2016). PAV decreased by 0.95% in the evolocumab group, whereas it increased by 0.05% in the placebo group (P<0.001). The proportion of patients achieving plaque regression was also significantly higher in the evolocumab group (64.3% vs. 47.3%, P<0.001 for PAV).

#### 3.2.3 FOURIER: the first to confirm cardiovascular event reduction with PCSK9 inhibitor

Evolocumab was the first PCSK9 inhibitor to demonstrate cardiovascular outcome benefits in a large-scale clinical trial. The FOURIER trial (Sabatine et al., 2017a) was a landmark, double-blind, randomized, placebo-controlled, multicenter Phase 3 study. The trial enrolled 27,564 patients with established ASCVD. Key inclusion criteria required that patients have a LDL-C level of ≥70 mg/dL or a non-high-density lipoprotein cholesterol (non-HDL-C) level of ≥100 mg/dL, while concurrently receiving an optimized background lipid-lowering regimen. The primary composite endpoint included cardiovascular death, myocardial infarction (MI), stroke, hospitalization for unstable angina, or coronary revascularization; the key secondary composite endpoint included cardiovascular death, MI, or stroke.

This study reported that in high-risk statin-treated patients, evolocumab reduced LDL-C levels by 59% and lowered the risk of major adverse cardiovascular events (MACE) by 15% (composite endpoint: cardiovascular death, MI, stroke, unstable angina, or coronary revascularization) (Sabatine et al., 2017a). These findings established the role of PCSK9 inhibitors in secondary prevention of ASCVD. Additionally, the FOURIER open-label extension study provided the longest follow-up data available for any PCSK9 inhibitor. Over more than 8 years of follow-up, LDL-C levels remained consistently and markedly low, with early initiation of evolocumab therapy associated with further reduction in long-term cardiovascular risk (Gaba et al., 2023). Furthermore, in patients with ASCVD, achieving sustained very low LDL-C levels below 20 mg/dL (<0.5 mmol/L) with evolocumab was linked to a reduced risk of cardiovascular events, without raising significant safety concerns (Gaba et al., 2023).

#### 3.2.4 VESALIUS-CV: the first to confirm cardiovascular benefit of PCSK9 inhibitor in primary prevention population

The VESALIUS-CV trial is a phase 3, double-blind, randomized, placebo-controlled global study (Bohula et al., 2026). It enrolled 12,257 patients with atherosclerosis or high-risk diabetes mellitus and no history of myocardial infarction or stroke. The study aimed to assess whether evolocumab, through LDL-C lowering, could reduce MACE in a primary prevention population. After a median follow-up of 4.6 years, evolocumab significantly lowered the incidence of 3-point MACE (a composite of death from coronary heart disease, myocardial infarction, or ischemic stroke, 6.2% vs. 8.0%; P < 0.001) and 4-point MACE (a composite of 3-point MACE or ischemia-driven arterial revascularization, 13.4% vs. 16.2%; P < 0.001) compared with placebo, with no significant safety differences. These results demonstrate that evolocumab reduces the risk of first cardiovascular event in this primary prevention cohort. By extending the benefits of potent LDL-C lowering to individuals without prior cardiovascular events, this finding challenges the traditional paradigm that such aggressive lipid-lowering therapy is reserved solely for secondary prevention. It establishes a new benchmark for early intervention, potentially redefining treatment thresholds for high-risk primary prevention patients.

#### 3.2.5 HAUSER-RCT: the first to evaluate cognitive safety with FH of PCSK9 inhibitor

Evolocumab is also the first PCSK9 inhibitor to have its cognitive effects systematically evaluated in pediatric patients with FH. The HAUSER-RCT trial (Santos et al., 2020) was a phase 3, randomized, double-blind, placebo-controlled study conducted in children and adolescents aged 10–17 years with heterozygous FH. Over 24 weeks of treatment, evolocumab significantly reduced LDL-C levels by approximately 38% compared with placebo, with no significant differences observed in cognitive function as assessed by standardized neuropsychological tests. Safety profiles were similar between groups, and no treatment-related adverse effects on neurocognitive development were reported.

Although cognitive function was an exploratory endpoint in this trial, these findings support the cognitive safety of evolocumab in the pediatric FH population and provide important evidence for its use in younger high-risk patients (Santos et al., 2020). Given that atherosclerotic processes begin in childhood for patients with FH, the ability to intervene safely during this developmental window has profound implications for lifelong cardiovascular risk reduction.

## 4 Comparison of PCSK9 inhibitors in China

China bears a substantial and growing burden of ASCVD all over the world, and the accessibility and affordability of PCSK9 inhibitors in the Chinese healthcare system represent a distinctive clinical scenario that is underrepresented in the English-language literature. Moreover, China has seen the rapid emergence of domestically developed PCSK9 inhibitors, with four novel monoclonal antibodies (tafolecimab by Innovent Biologics, approved in 2023; ebronucimab by Akeso Biopharma, approved in 2024; ongericimab by Junshi Biosciences, approved in 2024; and recaticimab by Hengrui Pharma, approved in 2025) now available alongside the imported agents evolocumab and alirocumab (Sanofi, first approved globally by the FDA in 24 July 2015 and approved in China in 2019, while scheduled for withdrawal from the Chinese market in 2025), as well as the small-interfering RNA (siRNA) agent inclisiran (Novartis, first approved globally by the EU in 2020 and approved for marketing in China in 2023), making China one of the most competitive markets for PCSK9-targeted therapies globally. Across diverse patient populations, these PCSK9 inhibitors have demonstrated potent lipid-lowering efficacy along with favorable safety and tolerability profiles in clinical studies. Data concerning their respective market availability, regulatory status, pharmacokinetic differences, clinical trial comparisons, potential advantages and disadvantages, and strength of outcome evidence are detailed in Table 1.

**TABLE 1:** Approved PCSK9 inhibitors in China.

Characteristic	PCSK9 monoclonal antibody						siRNA	Reference
	Evolocumab	Alirocumab	Recaticimab	Ebronucimab	Ongericimab	Tafolecimab	Inclisiran	
First global approval	2015/07/1 (EC)	2015/07/24 (FDA)	—	—	—	—	2020/12/9 (EC)	​
Approval in China	2018/07/31	2019/12/26 (The supply has been discontinued in August 2025)	2025/01/10	2024/09/26	2024/10/11	2023/08/16	2023/8/22	​
Antibody type	Fully human IgG2 monoclonal antibody	Humanized IgG1 monoclonal antibody	Fully human IgG1 monoclonal antibody	Fully human IgG1 monoclonal antibody	Fully human IgG4 monoclonal antibody	Fully human IgG2 monoclonal antibody	—	​
Dosage regimen	140 mg Q2W or 420 mg Q4W	75 mg or 150 mg Q2W	150 mg Q4W or 300 mg Q8W	150 mg Q2W or 450 mg Q4W	150 mg Q2W or 300 mg Q4W or 450 mg Q4W	150 mg Q2W or 450 mg Q4W or 600 mg Q6W	284 mg Q6M (After the first dose, it is administered again at 3 months and then every 6 months)	​
Half-life (t_1/2_)	11–17 days	17–20 days	22–27 days	11.8–14.2 days	4.5–6.5 days	26 days	9 h	Akeso Biopharma (2024); Innovent Biologics (2024); Jiang et al. (2024), Junshi Biosciences (2024); Amgen Inc (2025); Jiangsu Hengrui (2025); Novartis, 2025a; Sanofi (2025)
Bioavailability	72%	85%	70%	—	36.0%–55.5%	58%	85%	
Time to peak drug concentration	3–4 days	3–7 days	7–9 days	5 days	3–7 days	5–7 days	4 h	
Clearance	0.3	—	0.254–0.271	0.617	0.53–1.09	0.162	—	
Volume of distribution (L)	3.3	2.8–3.5	8.28–8.64	9.65–19.4	3.69–7.06	5.7	500	
ADA incidence	0.3%	5.5%	14.1%	9.4%	5.8%	10.9%	4.9%	
Key phase 3 trial	FOURIER	ODYSSEY	REMAIN-2	AK102-301	JS002-007	CREDIT-1	ORION	​
LDL-C reduction	59.2%–75%	44.5%–63.3%	53.4%–62.2%	59.13%–60.43%	66.2%	57.3%–65%	51.5%–57.0%	(Huo et al., 2023; Sun et al., 2024; Wilkinson et al., 2024; Zhang et al., 2024; Amgen Inc, 2025; Sanofi, 2025; Shao et al., 2025)
Decreased percent of atheroma volume	1.0% (78w)	1.21% (52w)	—	—	—	—	—	(Nicholls et al., 2016; Räber et al., 2022)
MACE reduction	15%	15%	—	—	—	—	26%	(Nicholls et al., 2016; Schwartz et al., 2018)
Injection-site reactions	3.2%	6.1%	3.8%	10.8%	3.6%–5.1%	7.3%	5.0%	(Huo et al., 2023; Akeso Biopharma, 2024; Innovent Biologics, 2024; Junshi Biosciences, 2024; Amgen Inc, 2025; Jiangsu Hengrui, 2025; Novartis, 2025a; Sanofi, 2025)

PCSK9, Proprotein convertase subtilisin/kexin type 9; siRNA, small interfering RNA; EC, European commission; FDA, food and drug administration; ADA, anti-drug antibody; LDL-C, low-density lipoprotein cholesterol; MACE, major adverse cardiovascular event.

### 4.1 Mechanistic differences

Currently, two classes of PCSK9 inhibitors are clinically available: fully human monoclonal antibodies and siRNA-based therapeutics. While both effectively reduce PCSK9 activity, their mechanisms of action differ. Monoclonal antibodies, such as evolocumab, alirocumab, bind to circulating PCSK9 in the plasma and block its interaction with the LDLR on hepatocytes. This prevents PCSK9-mediated LDLR degradation, allowing more receptors to recycle to the cell surface and thereby enhancing hepatic uptake and clearance of LDL-C (Seidah and Prat, 2022). Clinically, evolocumab has been shown to achieve maximum suppression of free PCSK9 within 4 h of administration (Kasichayanula et al., 2018). The reductions of unbound PCSK9 levels at 1 week after evolocumab treatment were consistently around 90% regardless of background therapy (Blom et al., 2014).

In contrast, siRNA-based therapeutics such as inclisiran act intracellularly. Inclisiran is a double-stranded non-coding RNA that enters hepatocytes and is incorporated into the RNA-induced silencing complex (RISC). The RISC then uses the guide strand to bind and degrade *PCSK9* mRNA, thereby reducing PCSK9 protein synthesis (Carthew and Sontheimer, 2009). This “upstream” mode of inhibition enables sustained LDL-C lowering, with a single dose capable of suppressing PCSK9 levels over an extended period. However, factors such as intracellular delivery efficiency and saturation of the RISC complex limit the ability of siRNA therapy to completely silence PCSK9 expression (Gavrilov and Saltzman, 2012). At optimal doses and dosing intervals, inclisiran can inhibit approximately 80% of PCSK9 production (Ray et al., 2023).

### 4.2 Lipid-lowering and cardiovascular efficacy

The lipid-lowering efficacy of PCSK9 inhibitors has been consistently demonstrated in pivotal clinical trials, though the degree of LDL-C reduction varies across agents. Early studies including FOURIER and LAPLACE reported that evolocumab lowered LDL-C by approximately 59%–75% from baseline (Robinson et al., 2014; Sabatine et al., 2017a). The HUA TUO study further showed that evolocumab also improved other lipid parameters in Chinese dyslipidemia patients, such as total cholesterol and apolipoprotein B (apoB) (Tan et al., 2023). In the ODYSSE EAST trial, alirocumab reduced LDL-C by 56.0% from baseline to week 24 (Han et al., 2020). The CREDIT-1 study demonstrated that tafolecimab decreased LDL-C by 57.3%–65% (Huo et al., 2023). Similarly, in the AK102-301 trial, ebronucimab (150 mg Q2W) achieved a 60.43% reduction in LDL-C after 12 weeks (Zhang et al., 2024). The JS002-007 trial (2023) reported that ongericimab lowered LDL-C by 62.7% at week 12, with effects sustained through week 52 (Shao et al., 2025). Most recently, the REMAIN-2 study showed that all dosing regimens of recaticimab significantly reduced LDL-C; the 150 mg Q4W regimen yielded a 62.2% decrease, accompanied by meaningful improvements in non-HDL-C, apoB, and lipoprotein(a) (Sun et al., 2024). For siRNA-based therapy, the ORION program indicated that inclisiran—administered twice yearly (300 mg) after initial loading doses—maintained a persistent LDL-C reduction of approximately 50% (Wilkinson et al., 2024).

In addition to their potent LDL-C-lowering effects, certain PCSK9 inhibitors, such as evolocumab and alirocumab, have demonstrated clear cardiovascular benefits. Evolocumab currently possesses the most extensive cardiovascular outcomes evidence across both primary and secondary prevention settings. The FOURIER and FOURIER-OLE studies showed that evolocumab reduced the risk of MACE—including myocardial infarction, stroke, and coronary revascularization—by approximately 15% in patients with ASCVD, with a particularly pronounced reduction in myocardial infarction risk (approximately 27%) (Sabatine et al., 2017a). The VESALIUS-CV trial further confirmed these benefits in a high-risk primary prevention population (Bohula et al., 2026)^.^ For alirocumab, the ODYSSEY OUTCOMES trial demonstrated that in patients with recent acute coronary syndrome, treatment not only lowered MACE risk but also significantly reduced all-cause mortality (Schwartz et al., 2018). However, large-scale primary prevention cardiovascular outcomes data for alirocumab have not yet been published. The siRNA agent inclisiran has initiated cardiovascular outcomes programs including VICTORION-INITIATE (Novartis, 2025b) and VICTORION-2 PREVENT (Novartis, 2025c), both of which focus on patients with established ASCVD; results for cardiovascular endpoints are still pending. A primary prevention study, VICTORION-1 PREVENT, is ongoing in high-risk individuals without prior ASCVD and is expected to be completed in 2029 (Novartis, 2025d). For other PCSK9 inhibitors currently available in China, comprehensive cardiovascular outcomes data remain limited, and their long-term cardiovascular benefits require further validation in future studies.

Current evidence suggests that evolocumab, alirocumab, and inclisiran may exert beneficial effects on atherosclerotic plaque burden and stability. In the GLAGOV study (Nicholls et al., 2016), evolocumab treatment resulted in a significantly greater reduction in PAV compared with control therapy, with a higher proportion of patients achieving plaque regression. The PACMAN-AMI trial (Biccirè et al., 2023) showed that adding alirocumab to high-intensity statin therapy in patients with acute myocardial infarction produced “triple regression”—reduced PAV, decreased lipid content, and increased fibrous-cap thickness—in approximately one-third of participants, indicating substantial improvements in plaque composition. A recent single-center prospective pilot study of inclisiran in 35 patients with coronary artery disease (Trusinskis et al., 2025) reported a 46.7% reduction in the maximum lipid-core burden index (maxLCBI4 mm) after 15 months of treatment, supporting a potential plaque-stabilizing effect. The VICTORION-PLAQUE study (Revaiah et al., 2026) is aiming to evaluate the efficacy of inclisiran, when compared with placebo, on top of maximally tolerated statin therapy with or without other lipid-lowering therapy in reducing total coronary atheroma volume and atheroma compositional changes assessed by CCTA in patients diagnosed with nonobstructive coronary artery disease (NOCAD). The enrollment has been completed for the VICTORION-PLAQUE study, while the study itself remains ongoing with clinical results not yet available. To date, clinical evidence demonstrating plaque regression effects for other marketed PCSK9 inhibitors remains limited.

### 4.3 Safety and immunogenicity

Although currently marketed PCSK9 monoclonal antibodies are fully human antibodies, the incidence of anti-drug antibodies (ADAs) varies across agents. Available data indicate that the incidence of ADAs is relatively low with evolocumab (0.3%) (Amgen Inc, 2025), while higher rates have been reported for alirocumab (5.5%) (Sanofi, 2025), recaticimab (14.1%) (Jiangsu Hengrui, 2025), ebronucimab (9.4%) (Akeso Biopharma, 2024), ongericimab (5.8%) (Junshi Biosciences, 2024), and tafolecimab (10.9%) (Innovent Biologics, 2024).

Inclisiran, a chemically synthesized siRNA therapeutic, exhibits inherently low immunogenicity. Clinical data indicate that the incidence of ADAs with inclisiran is approximately 4.9% (Novartis, 2025a), and these antibodies are typically low-titer, transient, and do not significantly impact clinical efficacy (Ray et al., 2020).

Overall, PCSK9 inhibitors exhibit a favorable safety profile and good tolerability, although potential adverse reactions should still be monitored in clinical practice (Chaudhary et al., 2017). Injection-site reactions represent the most commonly reported adverse event for both monoclonal antibodies and siRNA-based agents, typically presenting as localized pain, erythema, or swelling. These reactions are generally mild to moderate, transient, and self-limiting (Kosmas et al., 2020). Other frequently observed adverse events include nasopharyngitis and upper respiratory tract infections.

## 5 Association of PCSK9 with other diseases

### 5.1 PCSK9 and diabetes: a complex bidirectional relationship

**FIGURE 3:**
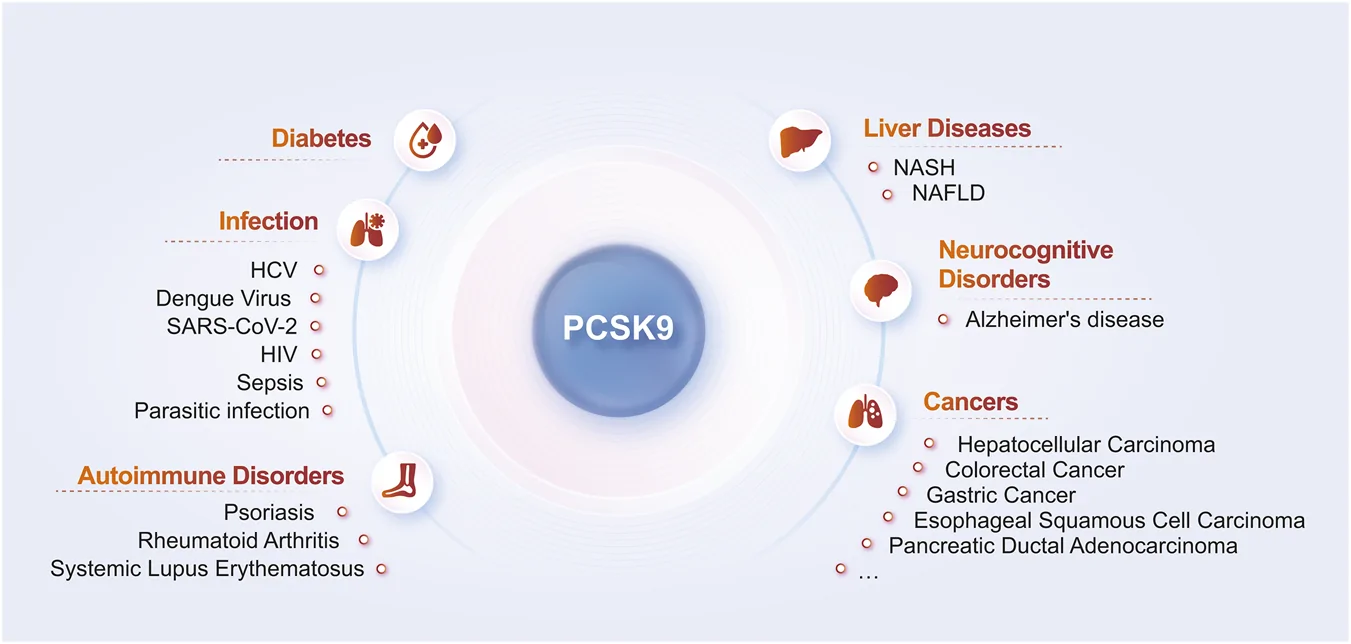
Extra-lipid-lowering effects of PCSK9 PCSK9, Proprotein convertase subtilisin/kexin type 9; HCV, Hepatitis C virus; SARS-CoV-2, severe acute respiratory syndrome coronavirus 2; HIV, Human immunodeficiency virus; NASH, Non-alcoholic steatohepatitis; NAFLD, Non-alcoholic fatty liver disease.

Beyond its well-established role in lipid metabolism, PCSK9 exhibits a bidirectional and intricate relationship with diabetes that extends beyond simple LDL-C–mediated pathways. At the mechanistic level, PCSK9 influences insulin secretion by modulating LDLR expression on pancreatic β-cell surfaces, and circulating PCSK9 levels correlate positively with fasting blood glucose, glycated hemoglobin (HbA_1c_), and insulin resistance in patients with type 2 diabetes mellitus (T2DM) (Momtazi et al., 2017) (Figure 3). Notably, genetic evidence reveals a potential trade-off between lipid and glucose homeostasis: LOF mutations in PCSK9 may carry a modestly increased risk of diabetes, whereas GOF mutations might confer reduced risk (Schmidt et al., 2017), suggesting that PCSK9 activity may influence glucose regulation through pathways yet to be fully elucidated. Clinically, this genetic complexity raises important therapeutic considerations. However, large-scale randomized controlled trials have provided reassuring evidence that PCSK9 inhibitors, such as evolocumab, exert no adverse effects on glycemic control and do not increase the risk of new-onset diabetes (Sabatine et al., 2017b). Furthermore, PCSK9 inhibitors significantly reduce the risk of MACE, including myocardial infarction, stroke, and cardiovascular death, in diabetic patients with established ASCVD (Sabatine et al., 2017b). Moreover, it’s reported that PCSK9 also accelerates both macrovascular and microvascular complications in diabetic patients through non-LDL-C-dependent pathways involving inflammation, thrombosis, and endothelial dysfunction (Punch et al., 2022; Gao et al., 2024). Together, these findings position PCSK9 as both a metabolic regulator and a therapeutic target in diabetes management, with PCSK9 inhibitors offering cardiovascular benefit without compromising glycemic safety.

### 5.2 PCSK9 in infectious diseases: from viral pathogens to sepsis

PCSK9 has emerged as a host factor that modulates susceptibility and outcomes across diverse infectious diseases, largely through its regulation of LDLR family members exploited by pathogens for cellular entry and the interaction between PCSK9 and innate immune signaling.

#### 5.2.1 Viral infections

##### 5.2.1.1 Hepatitis C virus (HCV)

*In vitro* experiment demonstrated that LDLR serves as a host entry factor for HCV infection of hepatocytes (Molina et al., 2007). In patients with HCV infection, serum PCSK9 levels are elevated and show a positive correlation with viral load, yet no correlation is observed with LDL-C levels, suggesting that PCSK9 may modulate HCV infection through mechanisms independent of lipid metabolism (Grimm et al., 2021) (Figure 3). Clinical studies reported that plasma PCSK9 levels rise significantly in HCV patients following treatment with direct-acting antivirals (Ichikawa et al., 2019), likely due to upregulation of LDLR after viral clearance. Therefore, caution is warranted when considering PCSK9 inhibitors in patients combined with active HCV infection, to avoid excessive LDLR upregulation that may theoretically facilitate viral particle assembly or cellular entry.

##### 5.2.1.2 SARS-CoV-2

Cholesterol-rich membrane microdomains facilitate the interaction between the SARS-CoV spike protein and angiotensin-converting enzyme 2 (Ren et al., 2006). Observational studies showed that plasma PCSK9 levels were elevated in sepsis patients with SARS-CoV-2 infection compared with non-COVID-19 sepsis patients (Mester et al., 2023) (Figure 3). Experimental evidence suggests that PCSK9 inhibition could reduce inflammatory cytokine levels, attenuate activation of the inflammatory cascade, and lessen lung injury and inflammation (Arsh et al., 2024), thereby may be helpful for preventing the development of acute respiratory distress syndrome. Furthermore, a double-blind, placebo-controlled, multicenter pilot trial (IMPACT-SIRIO 5) demonstrated that PCSK9 inhibition reduced the primary composite endpoint of death or intubation, as well as IL-6 levels, in severe COVID-19 patients compared with placebo (Navarese et al., 2023). In light of these findings, the administration of a single dose of a PCSK9 inhibitor might be considered upon confirmation of COVID-19 infection or observation of disease progression. However, more clinical evidence is needed to support this indication for PCSK9 inhibitor in future.

##### 5.2.1.3 Human immunodeficiency virus (HIV)

In a cross-sectional case-control study, circulating PCSK9 levels were approximately 65% higher in HIV-infected patients receiving antiretroviral therapy and correlated with endothelial dysfunction, contributing to accelerated atherosclerosis (Leucker et al., 2018) (Figure 3). Mechanistically, chronic inflammation and immune activation in HIV infection may upregulate PCSK9 expression, which in turn could exacerbate lipid abnormalities and accelerate atherosclerosis. Furthermore, some antiretroviral drugs have been implicated in dyslipidemia (Hemkens and Bucher, 2014), which may potentially synergize with PCSK9 to worsen metabolic and vascular health. These findings highlight PCSK9 may be treated as a promising biomarker and therapeutic target for reducing cardiovascular morbidity in the growing population of people living with HIV.

#### 5.2.2 Sepsis

PCSK9 deficiency reduces the risk of septic shock in both humans and mice (Walley et al., 2014) (Figure 3). Subsequent studies further showed that mice carrying PCSK9 LOF mutations have a lower incidence of septic shock and organ failure, whereas sepsis severity is exacerbated in transgenic mice overexpressing PCSK9 (Dwivedi et al., 2016). Plasma PCSK9 levels are greatly increased in sepsis and are highly correlated with the development of subsequent multiple organ failure (Boyd et al., 2016). Moreover, clinical observations indicated that patients carrying three specific PCSK9 LOF variants (R46L, A53V, and I474V) exhibited more than a 50% improvement in 1-year survival and a reduced risk of recurrent infections (Genga et al., 2018).

#### 5.2.3 Parasitic infection

Parasites depend on host-derived cholesterol for growth and development. Genetic evidence from malaria-endemic regions reveals that PCSK9 GOF mutations are associated with more severe disease progression in children (Arama et al., 2018), while PCSK9 LOF mutations correlate with reduced malaria-related mortality among Malian Children (Fedoryak et al., 2020). These findings suggest that modulation of PCSK9 activity may influence host susceptibility to parasitic infection (Figure 3), although relevant clinical evidence remains limited and warrants further investigation.

### 5.3 PCSK9 and liver disease: controversies and emerging directions

The role of PCSK9 in non-alcoholic fatty liver disease (NAFLD) remains controversial, with inconsistent clinical evidence currently available (Momtazi-Borojeni et al., 2022). Existing findings suggest that PCSK9 may influence NAFLD progression by modulating hepatic lipid accumulation and inflammatory responses. Preclinical studies have reported an inverse correlation between PCSK9 expression and the progression of NAFLD and non-alcoholic steatohepatitis (NASH) (Momtazi-Borojeni et al., 2022). In contrast, human studies indicate that hepatic PCSK9 expression correlates positively with the degree of hepatic steatosis, and circulating PCSK9 levels are also positively associated with NAFLD severity (Figure 3). Several clinical studies propose that PCSK9 inhibitors may improve NASH, potentially reverse hepatic steatosis, and help mitigate liver injury (Han et al., 2024).

### 5.4 PCSK9 and autoimmune diseases: shared inflammatory pathways

PCSK9’s proinflammatory properties position it as a common pathogenic thread across multiple autoimmune disorders.

#### 5.4.1 Psoriasis

Both clinical and preclinical studies indicate that local *PCSK9* mRNA levels in psoriatic lesions are elevated approximately five-fold. PCSK9 expression is also increased in the imiquimod-induced mouse model of psoriasis, whereas PCSK9-knockout mice fail to develop typical psoriasis-like lesions following imiquimod stimulation (Luan et al., 2019). These findings suggest that PCSK9 plays an important role in the formation of psoriatic plaques, likely mediated through interactions with the JAK and ERK signaling pathways (Figure 3). Importantly, PCSK9 levels in psoriasis patients are modulated by systemic therapies: methotrexate reduces PCSK9, while acitretin increases it, potentially influencing cardiovascular risk (Krahel et al., 2020). Mendelian randomization studies further support a causal link, showing that genetically mediated PCSK9 inhibition is associated with reduced psoriasis risk (Zhao et al., 2023).

#### 5.4.2 Rheumatoid arthritis (RA)

Plasma PCSK9 concentrations and the PCSK9/LDLR ratio positively correlate with the progression of atherosclerosis in RA patients (Arida et al., 2021). Serum PCSK9 levels are also positively associated with the Disease Activity Score for RA, Th17 cell proportion, Th17/Treg ratio, and C-reactive protein levels. Moreover, PCSK9 may serve as a predictive biomarker: in patients treated with conventional synthetic disease-modifying antirheumatic drugs (csDMARDs), PCSK9 levels decrease, and a larger reduction is linked to a higher likelihood of achieving disease remission (Meng et al., 2023). Previous studies have shown that PCSK9 promotes the secretion of multiple inflammatory cytokines (e.g., TNF-α and IL-1β), while PCSK9 monoclonal antibodies inhibit their production (Frostegård et al., 2021), suggesting that PCSK9 inhibitors may hold therapeutic potential in RA (Figure 3).

#### 5.4.3 Systemic lupus erythematosus (SLE)

Serum PCSK9 levels are significantly elevated in patients with SLE and are positively correlated with carotid intima-media thickness (IMT), an early marker of atherosclerosis (Fang et al., 2018). In a 5-year follow-up study of 539 SLE patients, high PCSK9 levels conferred a 2.51-fold increased MACE risk, independent of traditional cardiovascular risk factors (Mok et al., 2023). These findings suggest that PCSK9 may serve as a dual biomarker for disease activity assessment and cardiovascular risk stratification in SLE (Figure 3).

### 5.5 PCSK9 and neurocognitive disorders

Cholesterol homeostasis is fundamental to central nervous system function, contributing to myelination, synaptogenesis, and neurotransmission (Björkhem and Meaney, 2004). PCSK9 regulates LDL uptake in brain endothelial cells via LDLR modulation (Bell et al., 2023), and its activity within the central nervous system (CNS) can increase under pathological conditions when blood-brain barrier integrity is compromised (Dietschy, 2009; Zimetti et al., 2017). Growing evidence indicates that dysregulation of lipid metabolism—particularly cholesterol homeostasis—is closely linked to the pathogenesis of neurodegenerative diseases. In Alzheimer’s disease (AD), PCSK9 degrades or suppresses LDLR family members (LRP1, ApoER2), thereby impairing amyloid-β (Aβ) clearance and accelerating plaque deposition (Mazura et al., 2022). PCSK9 deletion in animal models reduces Aβ burden and improves cognitive function (Vilella et al., 2024), while cerebrospinal fluid PCSK9 levels in AD patients correlate positively with Aβ1-42, phosphorylated tau, and total tau (Zimetti et al., 2017; Courtemanche et al., 2018), highlighting its biomarker potential for early AD diagnosis. However, the therapeutic implications are complex. Mendelian randomization analyses and meta-analyses have indicated that long-term exposure to low PCSK9 levels may increase the risk of AD (Williams et al., 2020), creating a paradox that the inhibition beneficial for cardiovascular protection could potentially be detrimental for neurocognitive health. This duality underscores the need for tissue-specific and context-dependent therapeutic strategies.

### 5.6 PCSK9 and cancers: an emerging frontier in tumor biology and immunity

In recent years, the role of PCSK9 in cancer biology has gained considerable attention, with multi-omics analyses revealing variable PCSK9 mRNA expression across tumor types, that is elevated in hepatocellular carcinoma, colorectal, gastric, and esophageal cancers, but reduced in lung, kidney, and prostate cancers (Cerami et al., 2012; Gao et al., 2013). PCSK9 may promote tumor initiation and progression through three principal mechanisms (Figure 3). First, *in vitro* and *in vivo* studies suggested that PCSK9 disrupts intracellular cholesterol homeostasis and activates the PI3K/AKT and related signaling pathways, thereby enhancing tumor cell proliferation, invasion, and epithelial-mesenchymal transition (Wang et al., 2022). Second, *in vitro* studies indicated that PCSK9 can trigger the mitochondrial apoptosis pathway (upregulating Bax and downregulating Bcl-2) and induce endoplasmic reticulum stress-related apoptosis, which may paradoxically inhibit tumor growth under certain contexts (Xu et al., 2017). Third, and most notably, experimental evidence shows that PCSK9 directly binds to MHC-I and mediates its lysosomal degradation, thereby reducing tumor antigen presentation (Liu X. et al., 2020). Moreover, PCSK9 interacts with LDLR on CD8^+^ T cells, impairing T-cell receptor (TCR) recycling and downstream signaling, ultimately suppressing cytotoxic T-lymphocyte (CTL) function *in vitro* (Yuan et al., 2021). This immunomodulatory role has opened an exciting therapeutic avenue. In preclinical studies, PCSK9 gene knockout in mouse cancer cells significantly reduced or prevented tumor growth, independently of host LDLR status, and PCSK9 monoclonal antibody monotherapy delayed tumor progression and enhanced antitumor efficacy (Liu X. et al., 2020). Crucially, preclinical studies demonstrated synergistic antitumor efficacy when PCSK9 inhibitors were combined with immune checkpoint inhibitors (ICIs), with enhanced infiltration of CD8^+^ T cells and reduced regulatory T cells and myeloid-derived suppressor cells (Yang et al., 2023). Early-phase clinical trials are currently evaluating PCSK9 inhibitors/ICI combinations in lung cancer (Bao et al., 2024), which may represent a potential paradigm shift from PCSK9’s traditional lipid-focused therapeutics to cancer immunotherapy.

## 6 Novel strategies targeting PCSK9

### 6.1 Oral PCSK9 inhibitors

**FIGURE 4:**
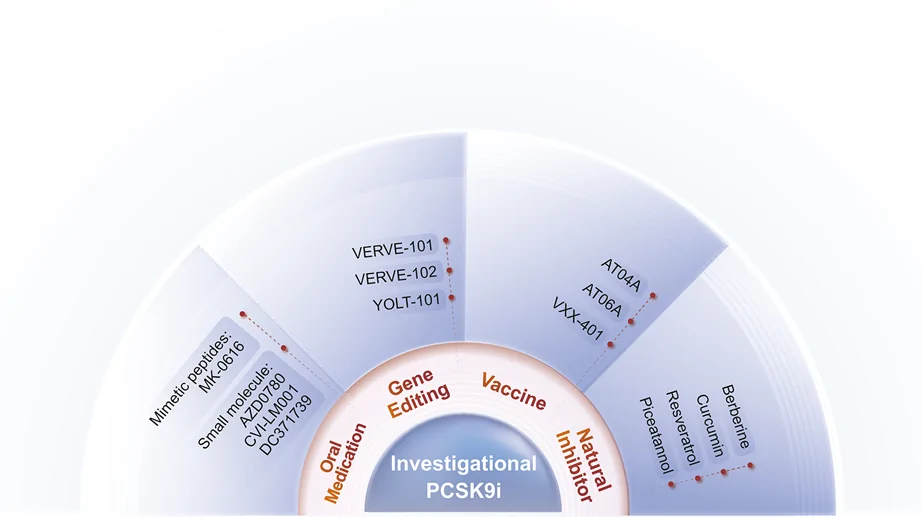
PCSK9-related interventional strategies PCSK9, Proprotein convertase subtilisin/kexin type 9.

Currently approved PCSK9 inhibitors require subcutaneous administration, which limits convenience and patient adherence. To address this, oral formulations are being developed as patient-friendly alternatives (Figure 4). MK-0616 (enlicitide decanoate) is a novel oral macrocyclic peptide that binds with high affinity to the LDLR-binding domain of PCSK9, thereby blocking PCSK9 from interacting with LDLR in human plasma (Corsini et al., 2025). Studies indicate that MK-0616 can reduce LDL-C by up to 66% from baseline (Tokgözoğlu et al., 2025). This agent is currently in phase 3 clinical development.

AZD0780 represents an oral small-molecule PCSK9 inhibitor with a distinct mechanism. Unlike siRNA or monoclonal antibodies, AZD0780 does not directly block PCSK9-LDLR binding; instead, it binds to the C-terminal domain of PCSK9 and inhibits lysosomal trafficking of the PCSK9-LDLR complex, allowing recycled LDLR to return to the hepatocyte surface. This unique mechanism lowers LDL-C levels by approximately 52% (Tokgözoğlu et al., 2025).

Other oral PCSK9 inhibitors are also under clinical investigation. CVI-LM001, an oral small-molecule transcriptional inhibitor of PCSK9, reduced serum PCSK9 levels by 36.4% (p < 0.001) after 10 days of treatment in normolipidemic healthy volunteers (Liu J. et al., 2020). It is currently in phase II clinical development. DC371739 is a dual transcriptional inhibitor of PCSK9 and ANGPTL3 that may retain lipid-lowering efficacy in patients with LDLR mutations (Ferri and Marodin, 2025). Mechanistically, DC371739 directly binds to hepatocyte nuclear factor 1α (HNF1α), a key upstream transcriptional regulator, thereby disrupting the transcriptional expression of both PCSK9 and ANGPTL3. This dual-target inhibition via HNF1α is distinct from existing small-molecule lipid-lowering agents.

Overall, oral PCSK9 inhibitors may offer a new lipid-lowering option with promising potential for short-term clinical translation. For patients who cannot tolerate injections or have poor treatment adherence, oral PCSK9 inhibitors may fill an important clinical gap.

### 6.2 Gene editing

Gene editing appears to be a promising strategy for achieving durable reductions in PCSK9 and LDL-C through direct genomic modification (Figure 4). Among available platforms, CRISPR/Cas9-based systems are favored for their efficiency and relative simplicity.

VERVE-101 is an investigational CRISPR base-editing therapy designed to introduce a single-nucleotide change in the PCSK9 gene, aiming to permanently suppress hepatic PCSK9 production and sustain LDL-C lowering (Hooper et al., 2024). In an open-label study, two patients with hypercholesterolemia receiving a 0.45 mg/kg dose achieved LDL-C reductions of 39% and 48%, respectively. However, interim analyses raised safety concerns: two participants experienced cardiovascular events, and others developed elevated alanine aminotransferase levels or thrombocytopenia requiring hospitalization (Hooper et al., 2024).

VERVE-102, a liver-selective base-editing therapy using GalNAc-LNP delivery, has shown improved safety. In a Phase 1b trial, 0.6 mg/kg reduced LDL-C by a mean of 53% (maximum 69%) with no treatment-related serious adverse events (Verve Therapeutics, 2025).

YOLT-101, the first PCSK9 base-editing therapy in clinical development in China, demonstrated a mean LDL-C reduction of 52.3% at 24 weeks in a Phase 1 trial, with no serious adverse events reported (Wan et al., 2026). Beyond PCSK9 targeting, CTX310 inactivates ANGPTL3 via CRISPR-Cas9, lowering LDL-C through an LDL receptor-independent mechanism. In a Phase 1 trial, 0.8 mg/kg reduced LDL-C by 48.9% and triglycerides by 55.2%, with no treatment-related serious adverse events (Laffin et al., 2025).

Collectively, these advances suggest ongoing clinical progress of gene editing approaches for lipid disorders, with next-generation candidates demonstrating encouraging efficacy and generally acceptable safety profiles.

### 6.3 PCSK9 vaccines

PCSK9 vaccines are designed to lower plasma LDL-C levels by stimulating the body to generate endogenous anti-PCSK9 antibodies, which block the interaction between PCSK9 and the LDLR (Figure 4). Current vaccine strategies under development include peptide-based vaccines, virus-like particle vaccines, and nanoparticle-based vaccines (Goksøyr et al., 2022; Fang et al., 2024; Sahebkar and Banach, 2024; Surma et al., 2024).

VXX-401 is a peptide-based vaccine that elicits targeted antibody responses against an epitope in the catalytic domain of PCSK9. VXX-401 elicited high-titer anti-PCSK9 antibodies in macaques that resulted in a 30%–40% reduction in plasma LDL-C relative to a control group of unvaccinated animals. Importantly, immunization with VXX-401 did not affect HDL-C levels and was not associated with any adverse events, such as chronic inflammation or uncontrolled autoimmunity against PCSK9 (Vroom et al., 2024). A network meta-analysis published in 2025 further demonstrated that VXX-401 achieved significantly greater LDL-C reduction than other PCSK9 vaccine candidates (e.g., rhPCSK9 VLP and HIT01) in non-human primate models (Muzaffar et al., 2025). AT04A and AT06A are peptide-based vaccines developed by AFFiRiS AG that have already undergone phase I clinical trials. However, AT04A exhibited a modest reduction in LDL-C, achieving a decrease of only 11%–13% (Zeitlinger et al., 2021).

As a long-acting strategy requiring infrequent administration, PCSK9 vaccines may represent a potentially promising approach for the management of dyslipidemia and ASCVD. Further clinical studies would be valuable to help validate their long-term efficacy, safety, and real-world value.

### 6.4 Natural PCSK9 inhibitors

Several natural compounds have been identified as potential PCSK9 inhibitors, modulating lipid metabolism through diverse mechanisms (Figure 4). Traditional Chinese medicinal herbs and plant-derived compounds, such as berberine, curcumin, resveratrol, piceatannol, sauchinone, lupin, quercetin, salidroside, ginkgolide, and extracts of Morus alba leaves, have demonstrated PCSK9-inhibitory activity (Han et al., 2024).

Although most of these compounds have shown encouraging efficacy in preclinical models, high-quality clinical data remain scarce. While berberine and silymarin have been evaluated in clinical studies and their lipid-lowering effects have been confirmed, the majority of other compounds are still in the preclinical or observational stage (Cicero et al., 2017; Fogacci et al., 2019). The primary challenges to clinical translation include poor oral bioavailability and the paucity of large-scale, well-designed randomized controlled trials (Cicero et al., 2017). Future research should focus on improving bioavailability via novel nano-formulation strategies and conducting adequately powered clinical trials to validate the lipid-lowering efficacy as well as potential cardiovascular benefits of these agents (Liu et al., 2025).

## 7 Discussion

PCSK9 is a key regulator of cholesterol metabolism, and its inhibitors have demonstrated potent LDL-C-lowering effects, highlighting their substantial potential for CVD prevention. Evolocumab, the first PCSK9 inhibitor approved worldwide, served as both a novel treatment for FH and a developmental blueprint for later monoclonal antibodies. The pivotal FOURIER trial first confirmed that evolocumab significantly lowers LDL-C and reduces the incidence of MACE in high-risk patients. Beyond lipid reduction, studies of evolocumab have also explored its capacity to promote atherosclerotic plaque regression, improve endothelial function, and guide therapy in pediatric FH populations—insights that have informed the broader advancement of PCSK9-targeted agents.

A range of PCSK9 inhibitors are now available in China, including the monoclonal antibodies evolocumab, alirocumab, tafolecimab, ebronucimab, ongericimab, and recaticimab, as well as the siRNA drug inclisiran. Clinical trials have confirmed their potent lipid-lowering efficacy, offering new therapeutic options for patients with hypercholesterolemia. However, for many of these agents, cardiovascular outcomes data remain limited, and evidence regarding their effects on atherosclerotic plaque progression and long-term safety in broader populations is still insufficient. Future studies are needed to clarify their cardiovascular protective potential and guide optimal clinical application.

Emerging evidence indicates that the biological functions of PCSK9 extend beyond cholesterol regulation. It also modulates other lipid components—such as triglycerides and lipoprotein(a)—and plays significant roles in diabetes, infections, liver disorders, autoimmune conditions, neurocognitive dysfunction, and cancers. These insights broaden the potential therapeutic applications of PCSK9 inhibitors beyond conventional lipid-lowering strategies.

Looking ahead, the development of PCSK9 inhibitors is poised to diversify and focus on patient-centric convenience. Novel strategies under investigation include oral macrocyclic peptides, gene-editing therapies, and vaccines. These approaches may offer longer-lasting efficacy and more convenient administration routes, which could potentially improve treatment adherence and clinical outcomes. In summary, while much has been elucidated regarding PCSK9 and its inhibitors, the continuous exploration of its diverse biology promises to unlock further therapeutic potential, moving from what we already know toward what remains to be discovered.
