# Correction: A narrative review of PCSK9 inhibitors: from evolocumab to novel discoveries

**DOI:** 10.3389/fphar.2026.1964681

**Published:** 2026-08-18

**Authors:** Hui-Hui Liu, Sha Li, Jian-Jun Li

**Affiliations:** 1 State Key Laboratory of Cardiovascular Disease, Fuwai Hospital, Heart Failure Center, National Center for Cardiovascular Diseases, Chinese Academy of Medical Sciences and Peking Union Medical College, Beijing, China; 2 State Key Laboratory of Cardiovascular Disease, Fuwai Hospital, Cardiometabolic Center, National Center for Cardiovascular Diseases, Chinese Academy of Medical Sciences and Peking Union Medical College, Beijing, China

**Keywords:** evolocumab, inhibitor, mechanism, pleiotropic effect, proprotein convertase subtilisin/kexin type 9

There was a mistake in Table 1 as published. In row “Antibody type”, Recaticimab was incorrectly described as “Fully human IgG1 monoclonal antibody”. The correct description is “Humanized IgG1 monoclonal antibody”.

The original version of this article has been updated.

